# Sources of information on HIV/AIDS used by adolescents and young people: A scoping review protocol

**DOI:** 10.1371/journal.pone.0340787

**Published:** 2026-02-10

**Authors:** Vanessa Carla do Nascimento Gomes Brito, Luzia Cibele de Souza Maximiano, Fernanda Belmiro de Andrade, Rogeria Moreira de Abrantes, Yenifer Lizeth Gañan Rojas, Jocellem Alves de Medeiros, Raissa Martins de Andrade, Elanna Nayele de Freitas Costa, Samuel da Silva Guedes, Beatriz de Oliveira Fernandes Felipe, Alexsandra Rodrigues Feijão

**Affiliations:** Department of Nursing, Federal University of Rio Grande do Norte, Natal, Rio Grande do Norte, Brazil; University of Saskatchewan, CANADA

## Abstract

**Objective:**

To describe the protocol for a scoping review of the main sources of information about HIV/AIDS used by adolescents and young people.

**Context:**

Studies have shown that adolescents and young people are increasingly using digital technologies as a source of information about HIV/AIDS. Although these digital technologies offer potential benefits to the educational process, their implementation and use are not always free from challenges and implications. In this sense, it is essential to investigate the reliability of the information sources accessed by adolescents and young people.

**Design:**

Scoping review protocol, conducted in accordance with JBI guidelines

**Method:**

This is a protocol for a scoping review to be conducted following the JBI guidelines, in three phases and using the following data sources: Scopus, Cumulative Index to Nursing and Allied Health Literature, Medical Literature Analysis and Retrieval System Online (MEDLINE), Scientific Electronic Library Online (SciELO), Latin American and Caribbean Literature in Health Sciences (LILACS), Web of Science and later, Google School, Capes Catalog of Theses and Dissertations, Theses Canada and USP Digital Library of Theses and Dissertations. The inclusion criteria are studies that address the theme, available in full, free of charge and without time or language restrictions. The exclusion criteria are opinion articles, letters to the editor and editorials. The results will be presented through discussions, tables, graphs and percentages. Expected results: The results are expected to reveal the main sources of information used by adolescents and young people regarding HIV/AIDS, allowing the identification of their preferences regarding the means and formats of access to content. This protocol is available for access through the following electronic address: https://osf.io/aunkh/ with https://doi.org/10.17605/OSF.IO/AUNKH.

**Conclusion:**

Data analysis will help to identify potentialities and limitations in strategies for accessing information about HIV/AIDS by adolescents and young people. This scoping review can foster knowledge about information needs, guiding the creation of more effective content, supporting health decision-making and strengthening communication between professionals, users and institutions, contributing to the access of reliable data.

## Introduction

According to the World Health Organization (WHO), adolescence is defined as the period between 10 and 19 years of age, while youth encompasses individuals aged 15–24 years. These stages of human development are marked by significant changes in the body, emotions and behavior, especially in sexuality. In this perspective, access to reliable and evidence-based sources of information about HIV/AIDS becomes essential to guide decisions, promote self-care and prevent infections among adolescents and young people, who make up a group with specific vulnerabilities to STIs, especially HIV [1,2].

Updated data from 2023 indicate that young people between the ages of 15–24 accounted for 23.4% of new HIV diagnoses in Brazil. The progression of HIV infection to AIDS within this age group is also a matter of concern. Between 2012 and 2022, 52,415 cases of young people progressing from HIV to AIDS were recorded, highlighting this group’s vulnerability to infection and disease progression [3].

Information sources can take on different formats, from physical records to digital content, and as they evolve, they expand the possibilities for communication, education, and self-care, especially among adolescents and young people living with HIV [4]. In the context of health, especially when faced with complex issues such as HIV/AIDS infection, access to reliable information becomes essential for raising awareness, preventing and coping with the disease [5].

Although there are initiatives aimed at HIV/AIDS education, knowledge is still limited about which sources of information are most used by adolescents and young people, how these contents are accessed and perceived, and what gaps persist. In this context. Accordingly, HIV/AIDS education for adolescents and young people is essential not only to enhance knowledge on prevention, but also to foster safe health behaviors in a contextually appropriate manner.

Different sources of information — including social media, health professionals, peers, and scientific literature — have distinct credibility and accessibility characteristics, shaping how young people understand and apply the content they acquire. Therefore, the development of the proposed study is justified by the importance of better identifying and comprehending all the various dimensions involved in adolescents’ and young people’s access, use, and perception of HIV/AIDS information sources.

### Background

HIV/AIDS infection continues to be a major public health problem, especially among adolescents and young people, an age group that presents specific vulnerabilities related to the onset of sexual life, the search for identity, peer influence and difficulty in accessing health services.

Therefore, access to age-appropriate, high-quality information is essential for health promotion, infection prevention, and treatment adherence. The decision to develop a scoping review protocol aligns with the understanding that this is the most suitable study design in this context, compared to other evidence synthesis approaches, as it seeks to systematically map the literature on the topic in a broad manner, with the aim of identifying key concepts, examining the scope of existing knowledge, and highlighting potential gaps.

Adolescence and young adulthood are stages of the life cycle characterized by profound biopsychosocial transformations that directly influence health-related behaviors. In the context of HIV/AIDS, this population is regarded as a priority due to their heightened vulnerability to infection, associated with factors such as early sexual initiation, limited access to health services, and exposure to information of varying reliability. Despite the importance of this issue, there is a lack of synthesized evidence systematizing knowledge about the sources of information adolescents and young adults rely on to understand and prevent HIV/AIDS, which constrains the development of more effective educational strategies.

A scoping review was deemed the most appropriate methodological approach to address this gap, as it allows for a comprehensive and systematic mapping of the extent, nature, and characteristics of the existing literature. Unlike systematic reviews, which focus on answering specific questions regarding the effectiveness or impact of interventions, scoping reviews are suited to exploring emerging or less consolidated fields, enabling the identification of trends, key concepts, methodologies, and knowledge gaps. This approach provides a robust overview of the information sources accessed by this population.

Thus, the choice of a scoping review, rather than other types of review, is justified by the need to construct an initial and comprehensive synthesis of a still fragmented field of knowledge, thereby providing a foundation for future research, public policy planning, and the development of healthcare interventions that address the actual informational needs of adolescents and young people. This constitutes a fundamental step toward strengthening HIV/AIDS prevention strategies in this age group, grounded in an understanding of how knowledge is sought, transmitted, and appropriated.

### Objective

From this perspective, the following research question was raised: “What are the main sources of information about HIV/AIDS used by adolescents and young people?”.

## Methods

### Study

This is a scoping review protocol. A scoping review protocol is very important because it establishes the objectives, methodologies and reporting procedures of the scoping review in advance, ensuring transparency throughout the process [6]^.^ To ensure the quality of the study and its writing, this review will be in accordance with the JBI guidelines and PRISMA-ScR (Preferred Reporting Items for Systematic Reviews and Meta-Analyses Extension for Scoping Reviews) (S1 File) [6,7].

It is worth noting that the report of this protocol was guided by best practices and reporting items for the development of scoping review protocols [8].

### Registration

Initially, a planning protocol containing all the essential information for conducting the study was prepared and duly registered on an open science platform, the Open Science Framework. This protocol is available for access through the following electronic address: https://osf.io/aunkh/ with https://doi.org/10.17605/OSF.IO/AUNKH. This record details the research topic, the question of interest, the objective, the search strategy with the respective data sources, the descriptors used, the cross-referencing performed, and the criteria adopted for selecting the studies, as well as the description of the instrument used for data extraction.

The study originated from the following research question: “What are the main sources of information on HIV/AIDS used by adolescents and young people?” This question was schematized using the mnemonic PCC, as follows: the “P” represents the population (adolescents and young adults), the first “C” refers to the Concept, which refers to HIV/AIDS; and the second “C” refers to the Context, the sources of information, as per Table 1.

**Table 1:** Description and characterization of the PCC mnemonic used in the research.

**Population/Problem:** Adolescents and young people	The World Health Organization (WHO) defines adolescence as the age group that goes from 10 to 19 years old and is marked by intense physical, psychological and social transformations. During this period, individuals go through a significant transition process from childhood to adulthood, experiencing changes in their body, emotions and social interactions. The young adult phase, which according to the WHO goes from 20 to 24 years old, is characterized by a process of consolidation. Young adults are still adjusting to new responsibilities, such as entering the job market, forming more stable relationships and defining life goals.
**Concept:** HIV/AIDS	HIV (Human Immunodeficiency Virus) is a virus that attacks the immune system, especially defense cells called CD4 + T lymphocytes, weakening the body’s ability to fight infections and diseases. HIV infection can lead to AIDS (Acquired Immunodeficiency Syndrome), the most advanced stage of the disease, characterized by the appearance of opportunistic infections, tumors and other complications resulting from compromised immunity. Although there is still no cure, treatment with antiretrovirals allows people living with HIV to live a long, healthy and high-quality life, in addition to significantly reducing the transmission of the virus.
**Context:** Sources of information	Information sources are the means by which data, knowledge or guidance on a given subject are obtained. They can be found in books, websites, videos, scientific articles, social networks, among others.

### Inclusion criteria

The inclusion criteria will be primary studies on HIV/AIDS information sources used by adolescents and young people, available in full, free of charge and without time or language limitations. Exclusion criteria will be review articles, opinion articles, experience articles, letters to the editor, editorials, commentaries, abstracts and protocols.

### Sources of information

The study will follow the guidelines of the literature, with a search strategy carried out in three distinct phases. In the first phase, six data sources will be consulted, namely: Scopus, Cumulative Index to Nursing and Allied Health Literature (CINAHL), Medical Literature Analysis and Retrieval System Online (MEDLINE), Scientific Electronic Library Online (SciELO), Latin American and Caribbean Literature in Health Sciences (LILACS) and Web of Science.

The second phase of the search will be carried out on Google Scholar, CAPES Catalog of Theses and Dissertations, Theses Canada, USP Digital Library of Theses and Dissertations. Finally, the third and final phase involves searching the references of the studies selected in the previous phases.

### Search strategy

The following DeCS/MeSH descriptors and keywords will be used for the search: Adolescente, Jovem adulto, Adolescent, Young adult, HIV, Acquired Immunodeficiency Syndrome, Fonte de informação, Information Sources. These descriptors resulted in cross-references using the Boolean operators AND and OR, according to the following syntax: 1# AND 2# AND 3# (S2 File).

The use of indexed terms in DeCS/MeSH ensures the standardization of language and minimizes translation errors across studies. The search protocol will be reviewed by a specialist to assess the methodological rigor of the review, following the PRESS (Peer Review of Electronic Search Strategies) guideline. This process involves verification by another researcher or information specialist to validate that the search strategy is accurate, comprehensive, and free of errors.

This strategy contributes to the transparency and rigor of the review, as it allows another researcher to verify whether the descriptors, Boolean operators (AND/OR), truncations (*), and if the combinations are appropriately applied.

To ensure consistency of results, the studies will be collected in a single day, using the Federated Academic Community (CAFe) of the Journal Portal of the Coordination for the Improvement of Higher Education Personnel (CAPES), which provides free access to some articles, journals and data sources.

### Selection of sources of evidence

The results obtained from the data sources will be exported to the Microsoft Excel research support tool. In this platform, two researchers will conduct the initial selection of studies independently and blindly, based on the analysis of their titles and abstracts. After ensuring agreement between the two researchers regarding the selected studies, these will be examined in full and included in the sample.

In the event of disagreements, a third invited researcher will be consulted to resolve the issue. The entire process follows the PRISMA-ScR flowchart and will be duly documented to allow detailed monitoring of the selection process.

### Inclusion of additional sources

After data collection in the first and second phases, the researchers will analyze the references of the studies initially collected to add new studies to the sample, respecting the recommended saturation.

### Second stage: full-text screening

The studies in the sample will be divided equally among the researchers so that they can read them in full and extract study variables.

### Data extraction

Study data will be collected using an extraction tool adapted from the model proposed by JBI for scoping reviews [7].

Data will be stored in an electronic spreadsheet linked to Microsoft Excel and organized according to the variables described in data collection instrument (S3 File) including identification number, data source, title, language(s), country of publication, authors, year of publication, methodological aspects and sources of information, level of evidence and application.

A pilot test of data extraction will be performed on a random sample of 25 studies to ensure that the extracted variables meet the purpose of the review. The entire research team involved in this review will examine the instrument according to the eligibility criteria and the variables selected for the study. Discrepancies will be discussed and modifications to the instrument variables will be made. The data collection will take place between December 2025 and February 2026, the final data review and analysis stage will be in March 2026 and the preparation and publication of the manuscript will take place in April 2026 (S4 File).

### Consultation with experts

To align the scoping review with best methodological practices and validate its findings, a phase of consultation with experts and stakeholders will be incorporated. This consultation will involve healthcare professionals, HIV/AIDS researchers, educators, and representatives of organizations working with adolescents and young people. Experts will be invited to review the proposed categories for classifying information sources, assess the relevance and clarity of credibility and accessibility criteria, and provide suggestions to enhance the interpretation of the findings. The consultation will be conducted via electronic forms, ensuring a systematic record of contributions and integrating their recommendations into the final synthesis, thereby strengthening the validity and practical applicability of the review.

### Evidence analysis

Ultimately, after applying all eligibility processes and criteria, the studies will constitute the sample. These will then be analyzed according to their level of evidence, following the JBI approach classification: level 1 – experimental studies; level 2 – quasi-experimental studies; level 3 – analytical observational studies; level 4 – descriptive observational studies; and level 5 – expert opinion and bench research [7]. This type of analysis will be carried out to fairly discuss the results of the studies according to the level of evidence presented. In addition, it will facilitate readers’ understanding of the level of evidence to base practice on (S3 File).

Although scoping reviews do not methodologically require a formal assessment of the quality of included studies, this limitation is acknowledged and addressed following the guidelines of the JBI. Accordingly, the review will adhere to the principles of transparency, clarity, and systematic reporting outlined in the manual, ensuring that data extraction and presentation capture essential information — such as study design, context, type of information source investigated, and characteristics of the target population — thereby providing a basis for critical appraisal of the body of evidence.

Additionally, gaps, trends, and methodological limitations identified in the studies will be discussed, in line with the JBI recommendations for scoping reviews. This approach will allow the findings to be contextualized without overstating the strength of the evidence, ensuring rigor in the synthesis process and providing a solid foundation for future research and the development of public policies aimed at HIV/AIDS prevention among adolescents and young people.

For this scoping review, an information source is defined as any medium, channel, or resource used by adolescents and young people to obtain knowledge about HIV/AIDS. The analysis of these sources will be guided by the concept of “reliability,” understood through two dimensions: (1) credibility — the legitimacy and recognition of the information provider; and (2) accessibility — the ease of access, clarity, and appropriateness of the content for the young audience.

Information sources will be categorized as social media, institutional sources, scientific literature, healthcare professionals, and peer networks.

### Ethical aspects

The research did not involve human beings; therefore, it was not necessary to submit it to the Research Ethics Committee.

## Results

**Fig 1:**
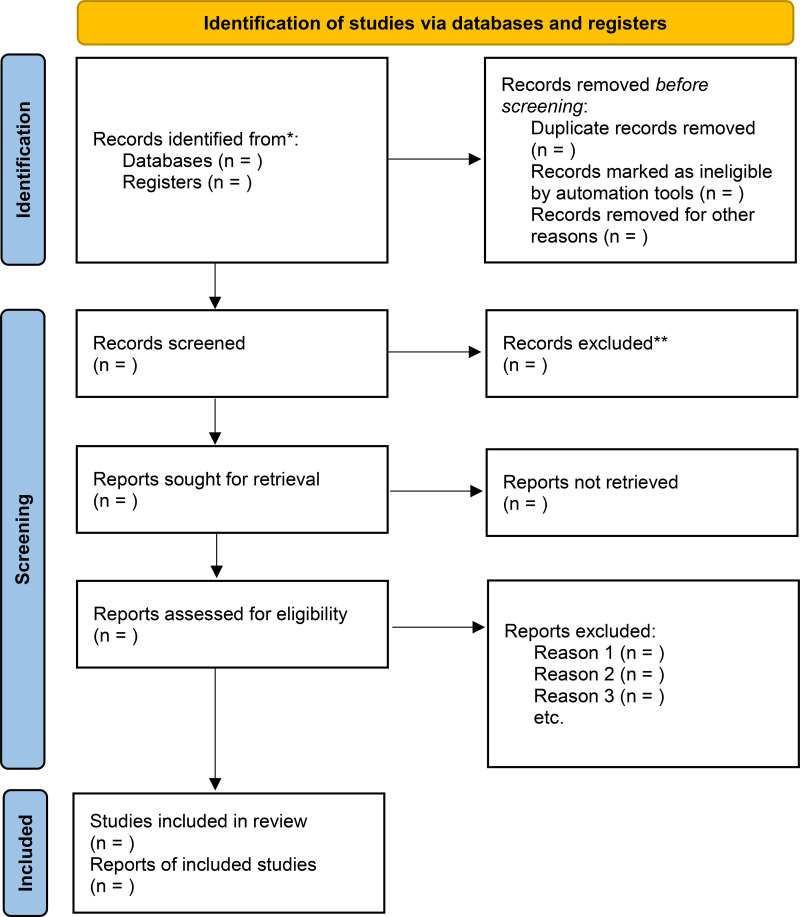
PRISMA 2020 flow diagram of study selection. Flow diagram illustrating the identification, screening, eligibility, and inclusion of studies in the scoping review. *Consider, if feasible to do so, reporting the number of records identified from each database or register searched (rather than the total number across all databases/registers). **If automation tools were used, indicate how many records were excluded by a human and how many were excluded by automation tools.

The results will be presented graphically through tables and boxes and in a descriptive manner, in order to answer the research questions, and the selected studies will be organized by figure according to the PRISMA 2020 flow diagram added to a narrative process of the findings (Fig 1). Flow diagram illustrating the identification, screening, eligibility, and inclusion of studies in the scoping review.

## Discussion

The review will provide comprehensive information and discuss the sources of information on HIV/AIDS used by adolescents and young people. It can further facilitate the identification of knowledge gaps in the literature and highlight areas where original research or more rigorous reviews are needed.

The results will also include a characterization of the studies comprising the sample. A table presented in figure format will allow a clear and organized visualization of the key information extracted from each study, including year of publication, country of origin, methodological design, target population, objectives, and main findings. This presentation will facilitate understanding of the profile of the included research and enable a more comprehensive and critical analysis of the scientific literature on the topic.

For this scoping review, an information source is considered any means, channel, or resource used by adolescents and young people to learn about HIV/AIDS. This definition encompasses digital sources (social media, websites, apps), institutional sources (educational materials, health campaigns), scientific sources (peer-reviewed articles), and professional sources (interactions with health workers and educators).

In addition to identifying the types of sources accessed, it is necessary to assess the concept of “reliability,” understood here as the perceived quality, credibility, and validity of the information obtained. For categorization purposes, reliability will be analyzed along two dimensions: (1) source credibility — the level of legitimacy or recognition attributed to the information provider (e.g., official organizations, healthcare professionals, digital influencers, peers); and (2) source accessibility — ease of access, clarity, and appropriateness of the content for the young audience. This framework will facilitate clearer comparisons across the included studies.

Social media are exemplified by platforms such as Instagram, TikTok, YouTube, and WhatsApp. This group of sources is characterized by everyday use, simplified language, and wide dissemination of content. Nevertheless, the credibility attributed to information from these platforms ranges from low to moderate, as it heavily depends on the profile of the information provider, the lack of quality control mechanisms, and the risk of misinformation circulation.

Institutional sources, which include government campaigns, educational materials from non-governmental organizations, and printed documents produced within the scope of public health policies, have a high level of credibility, as they are linked to official and socially recognized institutions. However, their accessibility can be classified as moderate, as the effectiveness of accessing this information depends on dissemination, distribution, and outreach strategies for the target audience, which are not always implemented uniformly.

Scientific sources, such as peer-reviewed articles and clinical guidelines, possess very high credibility due to methodological rigor and the review process that ensures the validity of the evidence presented. Yet, they exhibit low to moderate accessibility for adolescents and young people, due to barriers such as specialized technical language, the need for scientific literacy, and, in some cases, restricted access to publications because of financial or institutional limitations.

Healthcare professionals, including doctors, nurses, and health educators in general, constitute highly credible sources, supported by their technical training and the legitimacy of the scientific knowledge guiding their practices. Nevertheless, the accessibility to these sources tends to be moderate, as it depends on access to healthcare services and the quality of interactions between professionals and the young audience — factors that may be limited by structural and sociocultural barriers.

Ultimately, the network of peers and interpersonal sources, formed by friends, family, and community groups, show moderate credibility, based on personal experiences and empirical knowledge. Despite this limitation in terms of scientific validation, these sources are highly accessible because they are embedded in the daily lives of adolescents and young people, facilitating the spontaneous exchange of information within trusted and socially proximate spaces.

Among the limitations of the review, it is important to highlight the impossibility of accessing some studies by the researchers, due to the fact that they are paid, as well as the impossibility of individually evaluating the quality of the studies that made up the sample. In this perspective, it is recommended to carry out systematic reviews and meta-analyses with the objective of comparing methodologies and interventions used in the context of the study.

## Conclusion

Understanding the information sources on HIV/AIDS utilized by adolescents and young people is crucial for developing more effective health education strategies and promoting self-care. Mapping these sources allows for the identification of gaps in access to quality information, as well as opportunities to reinforce reliable and accessible channels. This insight also supports professionals, educators, and policymakers in creating content that is engaging, up-to-date, and tailored to the needs and realities of this population. Consequently, it contributes to the dissemination of trustworthy information and fosters the development of more informed and preventive behaviors among adolescents and young people.

## Supporting information

S1 File JBI PRISMA-ScR checklist. (DOCX)

S2 File Appendix for search strategies for each database. Detailed search strategies applied in each database.
(DOCX)

S3 File Data collection instrument. Structured instrument used to collect and organize data for the study.
(DOCX)

S4 File Scoping review steps schedule. Timeline of scoping review stages: data collection, review, and manuscript preparation.
(DOCX)
