# Supplementary material for: Sources of information on HIV/AIDS used by adolescents and young people: A scoping review protocol
Source: PLoS One. 2026 Feb 10;21(2):e0340787. doi: 10.1371/journal.pone.0340787 (PMC12890125; doi:10.1371/journal.pone.0340787)
Supplement: S2 File — Detailed search strategies applied in each database. (DOCX) [file pone.0340787.s002.docx]

| **SCOPING REVIEW** |
| --- |
| **Topic:** Sources of information on HIV/AIDS used by adolescents and young people: a scoping review |
| **Objective**: To map in the literature the sources of information on HIV/AIDS used by adolescents and young people over the years. |
| 1. **Research question** |
| What are the main sources of information on HIV/AIDS used by adolescents and young people?  **Population/Problem:** Adolescents and young adults  **Concept:** HIV/AIDS  **Context:** Sources of information |
| 1. **Identification of relevant studies** |
| **Search strategy:**  **1^st^ phase:**   - Database 1: Scopus - Database 2: Cumulative Index to Nursing and Allied Health Literature (CINAHL) - Database 3: Medical Literature Analysis and Retrieval System Online (MEDLINE) - Database 4: Scientific Electronic Library Online (SciELO) - Database 5: Latin American and Caribbean Literature in Health Sciences (LILACS) - Database 6: Web Of Science   **2^nd^ phase:**   - Google Scholar - CAPES Theses and Dissertations Catalog - Theses Canada - USP Digital Library of Theses and Dissertations   **3^rd^ phase:**   - Parallel search in references |
| **Indexed keywords or descriptors to be used in the databases:**  1#Adolescente, Jovem adulto  2#HIV,“Acquired Immune Deficiency Syndrome Virus”, “Acquired Immunodeficiency Syndrome”  3#Fonte de informação, Information Sources. |
| **Cross-referencing databases:**  **Scopus:** adolescent OR "Young adult" AND HIV OR “Acquired Immunodeficiency Syndrome” AND "Information Sources".  **CINAHL:** adolescent OR "Young adult" HIV OR “Acquired Immunodeficiency Syndrome” AND "Information Sources"  **Medical Literature Analysis and Retrieval System Online:** ((adolescent OR "Young adult" (HIV OR “Acquired Immunodeficiency Syndrome”) AND ("Information Sources").  **Scientific Electronic Library Online**: adolescent OR "Young adult" AND HIV OR “Acquired Immunodeficiency Syndrome” [Todos los indices] AND "Information Sources".  **Latin American and Caribbean Literature in Health Sciences:** (adolescente OR "jovem adulto” AND (HIV OR “Acquired Immunodeficiency Syndrome”) AND ("Fonte de informação).  **Web Of Science**: ((ALL=(adolescent OR "Young adult") AND ALL=(HIV) OR “Acquired Immunodeficiency Syndrome”) AND ALL=( "Information Sources") |
| 1. **Selection of studies** |
| - Inclusion criteria: primary studies that address the topic, available in full, free of charge and without a time limit. - Exclusion criteria: review articles, opinion articles, experience articles, letters to the editor, editorials, commentaries, abstracts and protocols. |
| **Selection strategy:**   - First, the studies will be selected based on a dynamic reading of the title and abstract by two researchers, independently. - The studies selected based on this reading of the title and abstract will be read in their entirety and the two researchers will be responsible for deciding whether to include or exclude each of them in the final sample. In the absence of a consensus between the two researchers, a third researcher will be invited to give the final opinion on the article. - Studies that are repeated in the databases will be counted only once and those that do not meet the inclusion criteria will be excluded. |
| 1. **Data collection** |
| **Mapping and data extraction:** This phase will be carried out using a data extraction tool.  **Consultation with experts:** Experts will be invited to review the proposed categories for classifying information sources, evaluate their relevance, clarify the criteria of accessibility and affordability, and offer suggestions to enhance the interpretation of the findings. |
| 1. **Report on the results obtained** |
| - The results will be presented through discussions, tables, graphs and percentages according to their specificities. |

**Instrument for data extraction**

| 1. **Identification of study** | |
| --- | --- |
| Identification number |  |
| Data source |  |
| Title of study |  |
| Language(s) |  |
| Country where the study was conducted |  |
| Author(s) |  |
| Year of publication |  |
| 1. **Methodological aspects** | |
| Objectives |  |
| Methods |  |
| Sample population |  |
| Age of participants |  |
| Type of study approach |  |
| Main results |  |
| 1. **Sources of information on HIV/AIDS** | |
| Television |  |
| Radio |  |
| School/ teachers |  |
| Friends/ colleagues/ community/ boyfriend/girlfriend |  |
| Parents/ guardians/ family |  |
| Digital media/ internet/ social networks |  |
| Magazines/ books/ pamphlets, newspapers |  |
| Others |  |
| 1. **JBI Level of evidence** | |
| Level 1 |  |
| Level 2 |  |
| Level 3 |  |
| Level 3a |  |
| Level 3b |  |
| Level 3c |  |
| Level 4 |  |
| 1. **Credibiliby and Reliability** | |
| Low |  |
| Moderate |  |
| High |  |
| Very High |  |
| 1. **Accessibility criteria** | |
| Low |  |
| Moderate |  |
| High |  |
| Very High |  |
