# Supplementary material for: Sources of information on HIV/AIDS used by adolescents and young people: A scoping review protocol
Source: PLoS One. 2026 Feb 10;21(2):e0340787. doi: 10.1371/journal.pone.0340787 (PMC12890125; doi:10.1371/journal.pone.0340787)
Supplement: S3 File — Structured instrument used to collect and organize data for the study. (DOCX) [file pone.0340787.s003.docx]

**Instrument for data extraction**

| 1. **Identification of study** | |
| --- | --- |
| Identification number |  |
| Data source |  |
| Title of study |  |
| Language(s) |  |
| Country where the study was conducted |  |
| Author(s) |  |
| Year of publication |  |
| 1. **Methodological aspects** | |
| Objectives |  |
| Methods |  |
| Sample population |  |
| Age of participants |  |
| Type of study approach |  |
| Main results |  |
| 1. **Sources of information on HIV/AIDS** | |
| Television |  |
| Radio |  |
| School/ teachers |  |
| Friends/ colleagues/ community/ boyfriend/girlfriend |  |
| Parents/ guardians/ family |  |
| Digital media/ internet/ social networks |  |
| Magazines/ books/ pamphlets, newspapers |  |
| Others |  |
| 1. **JBI Level of evidence** | |
| Level 1 |  |
| Level 2 |  |
| Level 3 |  |
| Level 3a |  |
| Level 3b |  |
| Level 3c |  |
| Level 4 |  |
| 1. **Credibiliby and Reliability** | |
| Low |  |
| Moderate |  |
| High |  |
| Very High |  |
| 1. **Accessibility criteria** | |
| Low |  |
| Moderate |  |
| High |  |
| Very High |  |
