# Supplementary material for: Sources of information on HIV/AIDS used by adolescents and young people: A scoping review protocol
Source: PLoS One. 2026 Feb 10;21(2):e0340787. doi: 10.1371/journal.pone.0340787 (PMC12890125; doi:10.1371/journal.pone.0340787)
Supplement: S4 File — Timeline of scoping review stages: data collection, review, and manuscript preparation. (DOCX) [file pone.0340787.s004.docx]

| Period | Activity | Responsable |
| --- | --- | --- |
| December 2025 – February 2026 | Data collection: systematic searches in databases, application of inclusion/exclusion criteria and data extraction (type of source, target audience, context, credibility, accessibility) | Researcher / Review team |
| March - 2026 | Revision and final data analysis: consistency verification, categorizing credibility and accessibility, descriptive and narrative synthesis, tables and graphics elaboration, identification of gaps. | Researcher/ Review team |
| April 2026 | Manuscript preparation and submission: writing sections (introduction, methodology, results, discussion, conclusion), critical review, formatting adjustments, tables and graphics insertion, submission preparation. | Researcher / Advisor |

**Timeline**
